# Identification of Novel NPRAP/δ-Catenin-Interacting Proteins and the Direct Association of NPRAP with Dynamin 2

**DOI:** 10.1371/journal.pone.0025379

**Published:** 2011-10-14

**Authors:** Carolina Koutras, Georges Lévesque

**Affiliations:** 1 Department of Psychiatry-Neurosciences, Faculty of Medicine, Laval University, Québec, Canada; 2 Neuroscience Unit, CHUL, Québec, Canada; McGill University, Canada

## Abstract

Neural plakophilin-related armadillo protein (NPRAP or δ-catenin) is a neuronal-specific protein that is best known for its interaction with presenilin 1 (PS1). Interestingly, the hemizygous loss of NPRAP is associated with severe mental retardation in cri du chat syndrome (CDCS), and mutations in PS1 cause an aggressive, early-onset form of Alzheimer's disease. Until recently, studies on the function of NPRAP have focused on its ability to modulate dendritic protrusion elaboration through its binding to cell adhesion and scaffolding molecules. However, mounting evidence indicates that NPRAP participates in intracellular signaling and exists in the nucleus, where it modulates gene expression. This apparent bifunctional nature suggests an elaborate neuronal role, but how NPRAP came to participate in such distinct subcellular events remains a mystery. To gain insight into this pathway, we immunoprecipitated NPRAP from human SH SY5Y cells and identified several novel interacting proteins by mass spectrometry. These included neurofilament alpha-internexin, interferon regulatory protein 2 binding factors, and dynamins 1 and 2. We further validated dynamin 2/NPRAP colocalization and direct interaction *in vivo*, confirming their *bona fide* partnership. Interestingly, dynamin 2 has established roles in endocytosis and actin assembly, and both of these processes have the potential to interface with the cell adhesion and intracellular signaling processes that involve NPRAP. Our data provide new avenues for approaching NPRAP biology and suggest a broader role for this protein than previously thought.

## Introduction

Neural plakophilin-related armadillo protein (NPRAP or δ-catenin) is a neuronal-specific protein that was first reported in a sequence search for plakophilin 1 homologous proteins [1]. Shortly after, clones encoding the human NPRAP were isolated from brain cDNA libraries as a presenilin-1 (PS1) biochemical partner [2], [3], [4], the then discovered and most commonly mutated protein in familial Alzheimer's disease (FAD) [5].

NPRAP was found to be exclusively expressed in the brain and neuroendocrine tissues [1], [3]. Supporting a neural-specific role for this protein, its gene, *CTNND2*, maps to a critical region on chromosome 5p15.2 that is deleted in cri du chat syndrome (CDCS) [6], [7]. CDCS features include a high-pitched cry at birth due to abnormal larynx development, low-set ears, microencephaly and severe psychomotor and mental retardation [8]. Symptom severity and the deletion size in the 5p chromosome vary, and, although another synaptic gene is located within the critical region, refined genotypic/phenotypic studies have revealed that the hemizygous loss of *CTNND2*, in particular, correlates with the severe mental retardation trait in CDCS patients [9]. Furthermore, *CTNND2* (−/−) null mice display synaptic plasticity and cognitive impairments [10].

NPRAP is highly conserved through metazoan evolution and, as a drosophila armadillo protein homolog, it belongs to the p120-catenin subfamily (for a recent review, see [11]). Members of this family also include p120-catenin, p0071, armadillo repeat protein deleted in velo-cardio-facial syndrome (ARVCF) and the plakophilins 1–3, which all share critical roles in cell-cell adhesion and signaling [12]. Their sequence encompasses a central domain composed of nine imperfect *arm* repeats that mediate protein-protein interactions. Moreover, NPRAP has long N- and C-terminal regions that comprise nuclear localization and export signals (NLS and NES), a coiled-coil domain, a (post-synaptic density protein-95 [PSD95], Drosophila disc large tumor suppressor [DlgA] and zonula occludens-1 protein [zo-1]) (PDZ) motif and several phosphorylation sites. NPRAP has been reported to interact with cell adhesion, anchorage and scaffolding proteins, including synaptic scaffolding molecule (S-SCAM) [13], densin-180 [14], plakophilin-related armadillo repeat protein-interacting PDZ protein (PAPIN) [15], erbin [16], cortactin [17], PSD-95, amiloride binding protein 1 (ABP) and cadherins [18]. Although NPRAP localizes to synapses, and studies in mouse primary neurons have suggested its involvement in a pathway regulating dendritic protrusion elaboration [19], many armadillo-like proteins and all of the p120-catenin family members have emerging roles in intracellular events [12]. Similarly, NPRAP has strong perikarya localization, along with a weak nuclear signal [1]. It has also been shown to regulate the rapsyn promoter at the neuromuscular junction through its binding to Kaiso, a bimodal DNA-binding protein [20]. In addition, we recently reported the requirement of NPRAP nuclear translocation for the regulation of genes implicated in cellular senescence, Alzheimer's disease and cancer (Koutras et al. 2011, JAD in press).

Surprisingly, either because NPRAP has no apparent link to the well-known γ-secretase activity of PS1 or because the function of this interaction has been difficult to assess using traditional approaches, its role has been poorly documented. Although research on NPRAP neuronal function has remained at an early stage over the past decade, the protein was brought to attention again as several groups reported its expression in prostate cancer cell lines [21], [22]. However, the mechanisms surrounding NPRAP regulation and function in epithelial cancerous cells have yet to be elucidated.

To date, the biological function of NPRAP in neurons is not known, and its participation in cell adhesion and signaling events has been studied separately. Using a combination of proteomic approaches, we sought to gain insight into this pathway by exploring the NPRAP interactome. We identified several novel NPRAP-binding proteins, including neurofilament alpha-internexin, interferon regulatory protein 2-binding factors 1 and 2 and Werner helicase-interacting protein 1. Interestingly, NPRAP was also found to bind the GTPases, dynamins 1 and 2, which are essential for endocytosis and implicated in signaling and actin cytoskeleton rearrangement. We further confirmed the direct interaction of NPRAP/dynamin 2 *in vivo* and their colocalization in neuronal SH SY5Y cells. These new findings strongly suggest the involvement of dynamin 2 in NPRAP-mediated intracellular signaling.

## Results

### NPRAP-interacting proteins

*Arm* repeats are shared by proteins with diverse cellular functions. The best-characterized example is β-catenin, whose crystal structure revealed its *arm* domain to be a single, relatively compact unit, which acts as a binding platform for different classes of proteins, including those involved in Wnt signaling [23], [24]. Therefore, we overexpressed a full-length NPRAP clone with its arm-repeat structure intact in human SH SY5Y cells and used an antigen purification strategy to identify NPRAP-interacting partners. Soluble proteins were extracted using a mild buffer, and the protein complexes were immunoprecipitated with anti-NPRAP monoclonal antibodies or mouse IgGs coupled to magnetic beads. The isolated proteins were separated according to their molecular mass under denaturing conditions and stained with Coomassie (1). All of the gel protein tracks, except for those corresponding to the IgG heavy and light chains bands, were excised and further analyzed by liquid chromatography coupled to tandem mass spectrometry (LC-MS/MS). The results, which correspond to two independent experimental samples and respective controls, were generated by Mascot (Matrix Science, UK) [25] and analyzed using Scaffold (Proteome Software, USA) set for stringent criteria. At a minimum confidence level of 95% for correct peptide and protein sequence identification, with at least two unique peptides identified, a given protein was considered as a putative NPRAP-binding partner if it was detected in both experimental samples and absent from the controls. In addition, keratins are common laboratory contaminants that were excluded from our results. A list of 14 proteins corresponding to these criteria and their respective gene ontology annotations are presented in Tables 1 and 2. These proteins include those that participate in gene repression and mRNA processing, as well as the structural neurofilament subunit alpha-internexin and a set of proteins that require energy from ATP or GTP hydrolysis to mediate DNA metabolism, actin polymerization regulation and endocytosis.

**Table 1:** A list of proteins identified by mass spectrometry as NPRAP-binding partners.

*Protein*	*Gene*	*Chromosome*	*Peptides#*	*Protein Coverage %*
Dynamin 2 isoform 2	*DNM2*	19p13.2	44	42
NPRAP/δ-catenin	*CTNND2*	5p15.2	36	41
Interferon regulatory factor 2-binding protein 2	*IRF2BP2*	1q42.3	23	51
Serine/arginine repetitive matrix 2 protein	*SRRM2*	16p13.3	15	12
Werner helicase-interacting protein 1 isoform CRA-c	*WRNIP1*	6p25.2	13	21
Hypothetical protein LOC80164	*FLJ22184*	19p13.2	12	15
GTP cyclohydrolase 1	*GCH1*	14q22.2	8	19
Alpha-internexin	*INA*	10q24.33	7	21
Poly(A)-binding protein, cytoplasmic 1	*PABPC1*	8q22.3	6	12
Interferon regulatory factor 2-binding protein 1	*IRF2BP1*	19q13.32	5	12
Dynamin 1 isoform 1	*DNM1*	9q34.11	5	18
ADP/ATP translocase 2	*SLC25A5*	Xq24	4	8.1
Enhanced at puberty protein 1	*IRF2BPL*	14q24.3	3	7.5
F-actin-capping protein subunit beta	*CAPZB*	1p36.1	2	8.7
Rho GTPase-activating protein 21	*ARHGAP21*	10p12.3	2	1.9

The protein and gene annotations are according to the Swiss Institute of Bioinformatics (SIB) [49] and the Hugo Gene Nomenclature Committee (HGNC) [50], [51]. Column 4 refers to the number (#) of unique peptides that matched the identified protein, whereas the corresponding percentage (%) of all of the amino acids detected in the protein sequence is presented in Column 5. Confidence level for correct protein sequence ≥95%.

**Table 2:** Functional annotations of NPRAP-interacting proteins.

*Protein*	*Molecular function*
Dynamin 2 isoform 2	Hydrolase (GTP→GDP)EndocytosisIntracellular synaptic vesicle/protein transport
NPRAP/δ-catenin	Signal transductionCell adhesionIntermediate filament-binding protein*
Interferon regulatory factor 2-binding protein 2	Transcriptional corepressor
Serine/arginine repetitive matrix 2 protein	mRNP complexes memberpre-mRNA processing*
Werner helicase interacting-protein 1 isoform CRA-c	ATPaseDNA helicase*
Hypothetical protein LOC80164	-
GTP cyclohydrolase 1	Hydrolase (GTP→7,8-DHNP-3′-TP, 7,8-NH2-3′-TP)
Alpha-internexin	Constituent of intermediate filament cytoskeleton
Poly(A)-binding protein, cytoplasmic 1	mRNA processing
Interferon regulatory factor 2-binding protein 1	Transcriptional corepressor
Dynamin 1 isoform 1	Hydrolase (GTP→GDP)EndocytosisIntracellular protein transport
ADP/ATP translocase 2	Amino acid transmembrane transporter activity
Enhanced at puberty protein 1	Zinc finger transcription factor
F-actin-capping protein subunit beta	Actin filament growth regulation
Rho GTPase-activating protein 21	GTPase-activating proteinActin filament polymerization regulation

Assignments were made using GeneTools [52] and Protein ANalysis THrough Evolutionary Relationships (Panther) [53], [54]. The scientific literature was used to validate all of the annotation hits and exclude recurrent errors arising from automated classification.

*Inferred from electronic annotation.

### Association of NPRAP with classical dynamins

**Figure 1:**
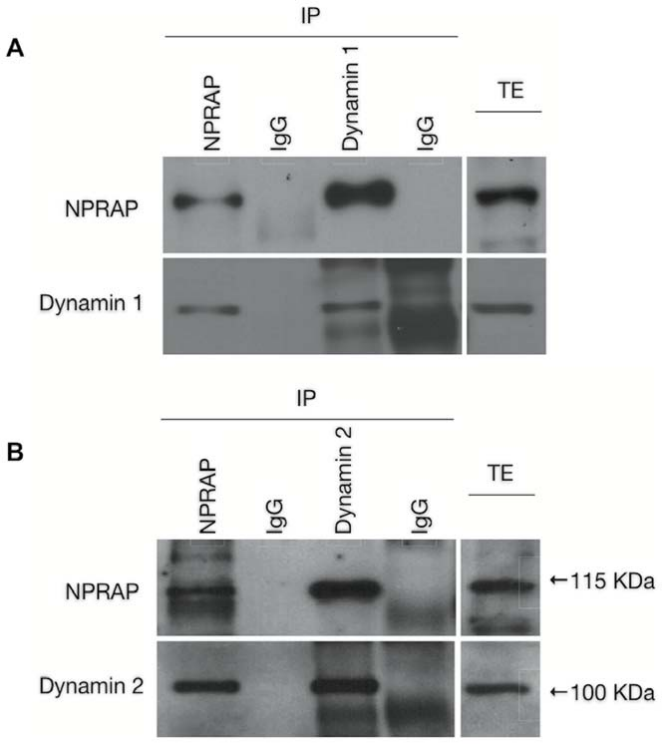
Co-immunoprecipitation of NPRAP and dynamins 1 and 2. As observed in the LC-MS/MS analysis, both dynamins co-precipitated with overexpressed NPRAP (A and B, lane 1, lower panels). Similarly, NPRAP was detected in the immunoprecipitations of dynamins 1 and 2 (A–B, lane 3, upper panels). Lanes 2 and 4 (A–B) correspond to mouse and goat serum IgG negative controls, respectively. Data are representative of at least three independent experiments. IP: immunoprecipitation; TE: total extract.

**Figure 2:**
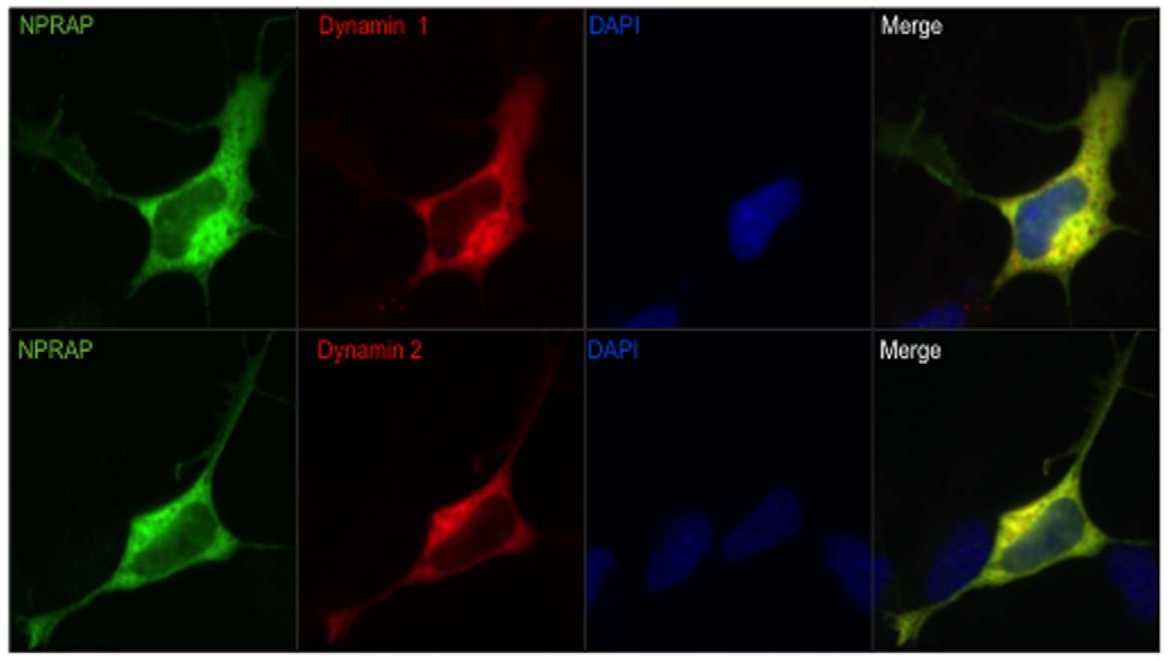
Dynamins colocalize with NPRAP. Immunolabeling studies showed that NPRAP and dynamin 1 or 2 colocalized in the perykarion of SH SY5Y cells. Note that this pattern seemed restricted to the cell body because the protrusions were labeled for NPRAP only, whereas a weak signal or lack of signal was observed for either dynamin 1 or 2 in those compartments. Images represent at least three independent experiments with similar immunofluorescent pattern results.

Among these novel partners, the classical dynamins 1 and 2 were of special interest because they participate in multiple cellular events, including the mechanochemical scission of vesicles required for membrane and cargo transport between different compartments in the cell [26]. To further validate our LC-MS/MS analysis and assess whether or not this NPRAP-dynamin 1/2 interaction was specific and of biological relevance, we immunoprecipitated NPRAP and dynamins 1 and 2 using specific antibodies. As confirmed by the western blot shown in Figure 1, NPRAP co-immunoprecipitated with both dynamins (*A* and *B*, lane 3, upper bands), and both dynamins co-immunoprecipitated with NPRAP (*A* and *B*, lane 1, lower bands). In agreement with the LC-MS-MS data, no signal for dynamins 1 and 2 could be detected in the mouse serum IgG controls, which was also true for NPRAP, thus refuting any possibility of a non-specific interaction. In addition, double-labeling immunofluorescence also revealed that NPRAP strongly colocalized with dynamins 1 and 2 in the perikaryon (Figure 2).

### NPRAP-dynamin 2 direct interaction

**Figure 3:**
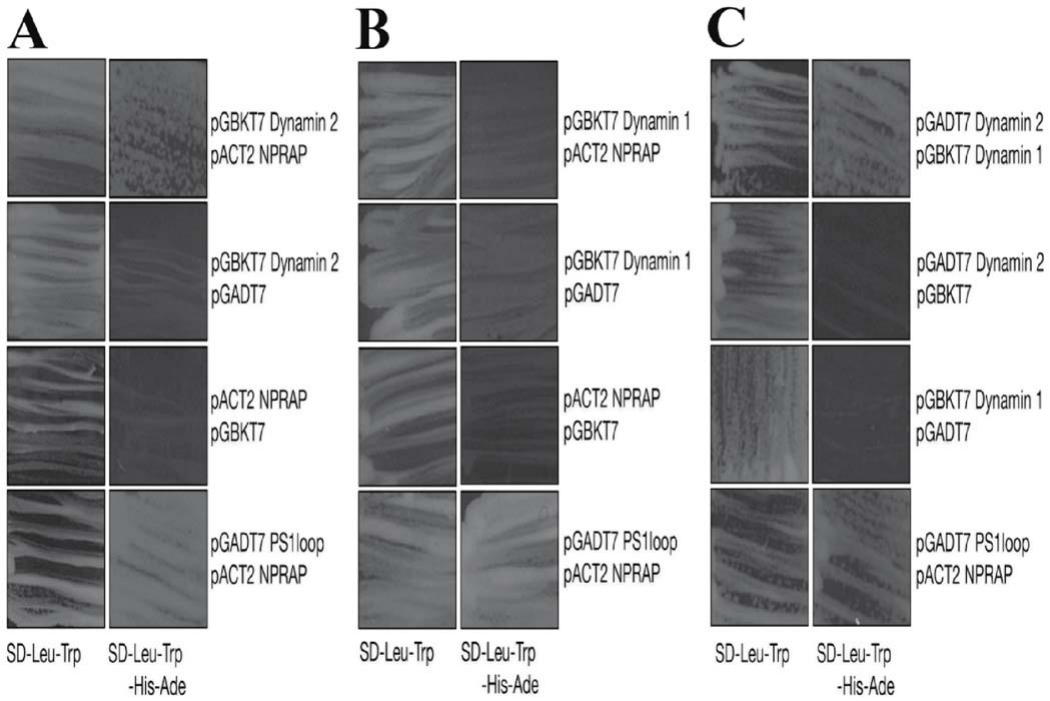
NPRAP binds dynamin 2 directly. Yeast transformants harboring plasmids that encode the hybrid “bait” and “prey” proteins survive in SD-Leu-Trp selective media (left columns). However, the survival in SD-Leu-Trp-His-Ade depends on the ability of the “prey” and “bait” to interact directly. Upon direct interaction, their hybrid activating and binding domains are brought close enough to reconstitute a functional transcription factor, which is needed to trigger the production of the above nutrients (right columns). Dynamin 2 interacted directly with NPRAP (A), whereas dynamin 1 did not (B). Dynamin 1 was functional in the system and interacted with dynamin 2, suggesting an NPRAP-dynamin 1 and 2 complex in the cells (C). Results represent five or more individual assays.

We next wanted to test whether this NPRAP-dynamin partnership resulted from a direct interaction or required the formation of a protein complex. Yeast two-hybrid screening [27] is a potent tool to verify direct protein-protein interactions *in vivo* because the reporter gene activation in this system only occurs if two hybrid proteins fused either to a DNA-binding domain or activating region are brought close enough to reconstitute a functional transcription factor. As seen in Figure 3-A, dynamin 2 clearly showed direct binding to NPRAP, whereas dynamin 1 did not (Figure 3-B). Even though an interaction between NPRAP and dynamin 1 could not be confirmed using this technique, further experiments revealed that dynamin 1 directly binds to dynamin 2 (Figure 3-C). Therefore, it is plausible that dynamin 2 bridges an interaction between dynamin 1 and NPRAP, thus forming a protein complex. Moreover, additional two-hybrid interaction analyses using a shorter NPRAP protein (amino acids 689–1285) suggest that the dynamin 2-NPRAP interaction required the last five *arm* repeats and the C-terminal region of NPRAP to occur (Figure S2).

## Discussion

Immunoprecipitation and LC-MS/MS are robust techniques that allow researchers to detect and distinguish multiprotein complexes from their biological milieu. When coupled to other approaches, such as the yeast two-hybrid system, they become an invaluable tool for studying and understanding protein function. Most of the NPRAP-interacting proteins that were previously reported in the literature were almost exclusively associated with the ability of NPRAP to induce dendrite growth. This has clearly limited research on NPRAP function. However, as NPRAP is an armadillo homolog and a PS1 partner, and as reports of its dynamic nucleocytoplasmic shuttling and role in gene regulation have emerged, it has become clear that NPRAP functions are not restricted to cell adhesion and protrusion elaboration. In an effort to address how a brain-specific protein evolved to exert such distinct, yet elaborate roles and to determine how the multiple roles of NPRAP are triggered and mediated, we identified 14 novel NPRAP-binding partners. To our knowledge, this is the first high-throughput proteomic analysis aimed at assessing the NPRAP interactome. Although we used a monoclonal antibody for the protein enrichment and nonspecific binding to IgGs was ruled out, “false” interactions cannot be fully excluded until individual validation using complimentary methods is performed. However, many of the interactions described herein may be stable (rather than transient and of lower affinity), because they survived the incubation and washing steps without the use of crosslinking agents. In addition, when proteins display similarity in sequence homology and molecular function, they have a greater probability of being specific interactors. Correspondingly, interferon regulatory factor 2-binding proteins 1 and 2 and enhanced at puberty 1 (encoded by the interferon regulatory factor 2-binding protein-like gene, *IRF2BPL*) are such candidates. Whereas enhanced at puberty 1 is a dual transcriptional regulator in the neuroendocrine system [28], the other two proteins seem to participate in the interferon pathway as co-repressors in an interferon regulatory factor 2-dependent manner [29]. Interestingly, we recently reported the involvement of NPRAP in transcriptional modulation, including the activation of interferon-inducible genes and the repression of several other targets (Koutras et al. 2011, in press). Remarkably, the above transcription factors were not the only proteins related to nucleic acid regulation identified in our study. Werner helicase-interacting protein 1 participates in DNA replication through its association with Werner syndrome ATP-dependent helicase, mutations of which result in genomic instability and premature aging [30]. Additionally, poly(A)-binding protein binds the poly(A) tail of mRNAs to regulate translation initiation, mRNA decay and silencing [31], whereas serine/arginine repetitive matrix 2 protein is a core member of the catalytic spliceosome that regulates the process by which introns are removed from precursor mRNAs [32]. All of the above-mentioned proteins reinforce a role for NPRAP in controlling gene expression. Interestingly, the only structural protein from the cytoskeleton detected in our analysis was the neurofilament subunit, alpha-internexin. This neuronal-specific intermediate filament exhibits axonal and dendritic localization [33] and has also been shown to induce neurite outgrowth in PC12 cells [34] and to mediate neurofilament anchorage to membrane-associated proteins and receptors [35]. Although armadillo proteins, such as β-catenin (but not p120-catenin), are believed to bind actin microfilaments [36], [37], this notion has been challenged [38], [39]. No studies have yet addressed if and how NPRAP may be associated with cytoskeleton components.

Lastly, a very interesting novel finding from our analysis was the identification of classical dynamins that were associated with NPRAP. Dynamins 1 and 2 are large GTPases that play an essential role in endocytosis, as they form a collar around the membrane necks of budding vesicles that rapidly triggers their scission upon GTP hydrolysis [26]. Contrary to general belief, dynamins are also functionally important in lamellipodia [40], phagocytosis [41], podosomes [42], and actin polymerization regulation [43]. The role of dynamins in tubulation and fission requires dynamic cytoskeleton rearrangements that are not restricted to the plasma membrane but also occur elsewhere in the cell, including in the trans-Golgi network where such rearrangement appears to involve dynamin 2-specific splice-variants [44].

In this study, NPRAP was found to colocalize and interact directly with dynamin 2 isoform 2. The increasing number of dynamin isoform variants encoded by the three human dynamin genes, together with their close sequence homology, makes it difficult to infer isoform-specific functional roles from the literature, as specific variants are not always mentioned. However, Cao et al. [45] studied the cellular distribution of several dynamin splice variants. The dynamin 2 “aa” variant in their report exhibited marked colocalization with the tubules of the Golgi apparatus and corresponds to the dynamin 2 sequence from our data, which also showed a similar perikaryal colocalization with NPRAP. We also identified the NPRAP interaction with Rho GTPase-activating protein 21, which appears to regulate actin dynamics at the Golgi [46] and is highly expressed in the brain. Moreover, F-actin-capping protein subunit beta is responsible for capping the barbed-ends of actin filaments, thus, regulating their growth [47], and was also identified in the study presented here.

Gu et al. [48] recently unveiled the much-anticipated dynamin-actin relationship by demonstrating that dynamin assembles directly on short actin filaments to promote their uncapping and subsequent polymerization in a GTPase-dependent mechanism. To some extent, these are interesting hints for addressing the possible roles of the NPRAP-dynamin 2 interaction in the perikaryon.

Overall, our search for NPRAP partners revealed proteins involved in every step of gene expression regulation and in membrane-mediated events, which are important for the buildup and transport of protein cargo and neurotransmitters, and the integrity of the synapse. In the context of a disease, such as Alzheimer's, where these processes are affected, it will be essential to investigate the relationship between PS1, NPRAP and the dynamins. We speculate an important biological link for dynamin 2 in NPRAP intracellular transport and signaling, and this is currently under investigation by our group. These extended putative roles for NPRAP were not predicted and might have important repercussions in the physiological role of the interaction of NPRAP with PS1.

## Materials and Methods

### Cells, transfection and plasmids

SH SY5Y human neuroblastoma cells (ATCC, USA) were cultured in Eagle's minimal essential medium (EMEM) plus Ham's F12 (50∶50) supplemented with 10% fetal bovine serum (FBS) and maintained at 37°C in a humidified atmosphere (5% CO_2_/95% air). Cells grown on 150-mm Petri dishes or glass coverslips were transfected using Lipofectamine™ 2000 reagent (Invitrogen, USA), according to the manufacturer's protocol. Dynamins 1 and 2 (human origin) were amplified by RT-PCR (as described below) using primer sets complimentary to the sequence of their 5′ and 3′ ends and designed to include restriction sites compatible with pCDNA3His (Invitrogen, USA), pGADT7 and pGBKT7 (Clontech, USA). pCDNA3His and pACT2 encoding full-length and short NPRAP human cDNAs and pGADT7/PS1loop (human origin) were from our laboratory collection.

### RNA extraction and RT-PCR

Total RNA from SH SY5Y cells was extracted using TRIzol® reagent (Invitrogen, USA), according to the manufacturer's protocol. Purified RNA (1 µg) was reverse-transcribed with 200 units of SuperScript® II reverse transcriptase (Invitrogen, USA) for 1 hour (37°C) following the supplier's recommended protocol. PCR reactions were performed with 2 µl of the RT product in a 50-µl mixture containing 0.4 mM dNTPs, 0.4 mM of each primer, 0.5% DMSO, Phusion GC buffer and 1 unit Phusion High-Fidelity DNA Polymerase (Finnzymes, Finland). The PCR conditions for both dynamins were 5 minutes (min) at 95°C, 30 cycles of 30 seconds (s) at 95°C, 30 s at 64°C, and 30 s at 72°C and 10 min at 72°C. The amplified genes were verified by restriction digestion profiling and sequencing.

### Immunoprecipitation and western blot

Confluent cell monolayers were washed twice with ice-cold PBS and lysed in 800 µl of STEN buffer (50 mM Tris [pH 7.6], 150 mM NaCl, 2 mM EDTA, 0.2% NP-40 and 0.5% Triton) supplemented with a protease inhibitor cocktail (Complete®, Roche). Cell lysates were transferred to Eppendorf tubes and incubated with agitation for 30 minutes (4°C). The lysates were then passed several times through a 25-gauge needle and centrifuged at 12,000 rpm for 10 minutes (4°C), and the cell debris was discarded. For each condition, 50 µl of the supernatant was retained for protein expression analysis, whereas the remaining sample was divided equally between experimental (antibody) and control tubes (mouse or goat IgGs). Immunoprecipitation was conducted using Dynabeads® Protein G (Invitrogen, USA), exactly as suggested by the manufacturer's protocol. The magnetic beads were complexed with the following antibodies: mouse anti-NPRAP for the LC-MS/MS analysis (3 µg; Santa Cruz, USA), mouse anti-NPRAP for the LC-MS/MS validation (3 µg; Abnova, Taiwan), goat anti-dynamin 1 (6 µg; Santa Cruz, USA), goat anti-dynamin 2 (6 µg; Santa Cruz, USA), or the equivalent amount of mouse or goat IgGs. The immunoprecipitated proteins were eluted in 30 µl of Laemmli buffer, boiled at 95°C for 5 minutes, separated by molecular weight in 10% SDS-PAGE and subjected to Coomassie staining (for LC-MS/MS) or transferred to a polyvinylidene difluoride (PVDF) membrane (for western blot). For the Coomassie stain, gels were washed, and the proteins were fixed in solution “a” (50% v/v ethanol and 10% v/v acetic acid in water) for 1 hour followed by an overnight incubation in solution “b” (50% v/v methanol and 10% v/v acetic acid in water). The solution was subsequently removed and gels were incubated in Coomassie stain solution (0.1% w/v Coomassie blue R250, 20% v/v methanol, and 10% v/v acetic acid in water) for 3 hours with gentle agitation. The gels were then destained in solution “b” by a series of washes until the bands were visible. Entire protein tracks were sliced and sectioned, excluding those sections corresponding to IgG light and heavy chains, and the tracks were then sent to the Proteomics Platform of the Quebec Genomics Center (Québec) for LC-MS/MS analysis (see below). For western blotting, the membranes were incubated in 5% non-fat milk in a Tris-buffered solution containing 0.1% Tween (TBS-T) for 30 minutes and then probed with mouse anti-NPRAP (1∶500; Santa Cruz, USA), goat anti-dynamin 1 (1∶250; Santa Cruz, USA) or goat anti-dynamin-2 (1∶250; Santa Cruz, USA) antibodies for 1 hour. After a series of washes in TBS-T, the membranes were re-probed with donkey anti-mouse or donkey anti-goat antibodies conjugated to horseradish peroxidase (1∶10000; Santa Cruz, USA) for one hour. The proteins were visualized using an ECL reagent (Perkin Elmer, USA).

### LC-MS/MS

All of the steps were performed according to the protocols and guidelines of the Proteomics Platform service. Briefly, in-gel protein digestions were performed on a MassPrep station (Micromass, USA). Peptides were separated by chromatography and eluted into a LTQ mass spectrometer (Thermo Fisher, USA) via a nanospray ionization. The MS/MS spectra generated were analyzed with Mascot (Matrix Science, UK) [25] for protein sequence identification. We used Scaffold (Proteome Software, USA) to select for NPRAP-interacting proteins using stringent criteria as specified in the Results section.

### Yeast two-hybrid assay

Competent *Saccharomyces cerevisiae* AH109 yeast cells (Clontech, USA) were co-transformed with pGADT7 and pGBKT7 vectors encoding hybrid “bait” and “prey” proteins. Controls for positive (pGADT7/PS1loop versus pACT2/NPRAP), negative (pGADT7 versus pGBKT7) and auto-activating (hybrid bait or prey versus non-hybrid DNA-activating or -binding domains) interactions were performed systematically. The procedure performed was according to the small-scale LiAc yeast transformation protocol described in the manufacturer's manual. Transformants harboring both bait and prey plasmids were selected on SD-Leu-Trp plates and restreaked onto SD-Leu-Trp-His-Ade medium for positive interaction selection.

### Microscopy

Cells on glass coverslips were fixed in 2% paraformaldehyde for 15 minutes, permeabilized in 0.1% saponin for 20 minutes and incubated with rabbit anti-NPRAP antibody (1∶1,000; Abcam, Taiwan) and goat anti-dynamin 1 (1∶50, Santa Cruz, USA) or goat anti-dynamin 2 (1∶50, Santa Cruz, USA) for one hour. The primary antibodies were in 2% BSA and 0.1% saponin in PBS. The cells were then washed twice with PBS and incubated with Alexa Fluor 488-conjugated goat anti-rabbit antibody or Alexa Fluor 682-conjugated donkey anti-goat (1∶250 in 0.1% saponin in PBS) (Invitrogen, USA). After 1 hour, the cells were washed and incubated for 15 minutes with DAPI (100 ng/ml; Sigma, USA) and then for 10 minutes with SlowFade® Gold (Invitrogen, USA). Controls for nonspecific staining were conduced accordingly. Coverslips were mounted on slides using Dako mounting medium (Dako, Denmark) and observed using epifluorescence microscopy (Carl Zeiss Axio Imager M2; Zeiss, USA). Images were acquired using an Axiocam MRm camera and the Axiovision Rel.4.8 software (Zeiss, USA).

## Supporting Information

Figure S1 **Coomassie stain patterns for protein samples from cells overexpressing NPRAP (lanes 2 and 4).** In lanes 3 and 5, the correspondent patterns for mouse serum IgG controls. Protein tracks from these gels were excised and further analyzed by LC MS/MS as described in the materials and methods section. MW: molecular weight.
(TIFF)

Figure S2 **NPRAP binding to dynamin 2.** A shorter NPRAP clone beginning in its fifth *arm* repeat (amino acid 650) also interacts directly with dynamin 2, strongly suggesting that their binding site is located after that repeat and within NPRAP's C-terminal sequence.
(TIFF)
