# Supplementary figures and images for: Identification of Novel NPRAP/δ-Catenin-Interacting Proteins and the Direct Association of NPRAP with Dynamin 2
Source: PLoS One. 2011 Oct 14;6(10):e25379. doi: 10.1371/journal.pone.0025379 (PMC3194794; doi:10.1371/journal.pone.0025379)

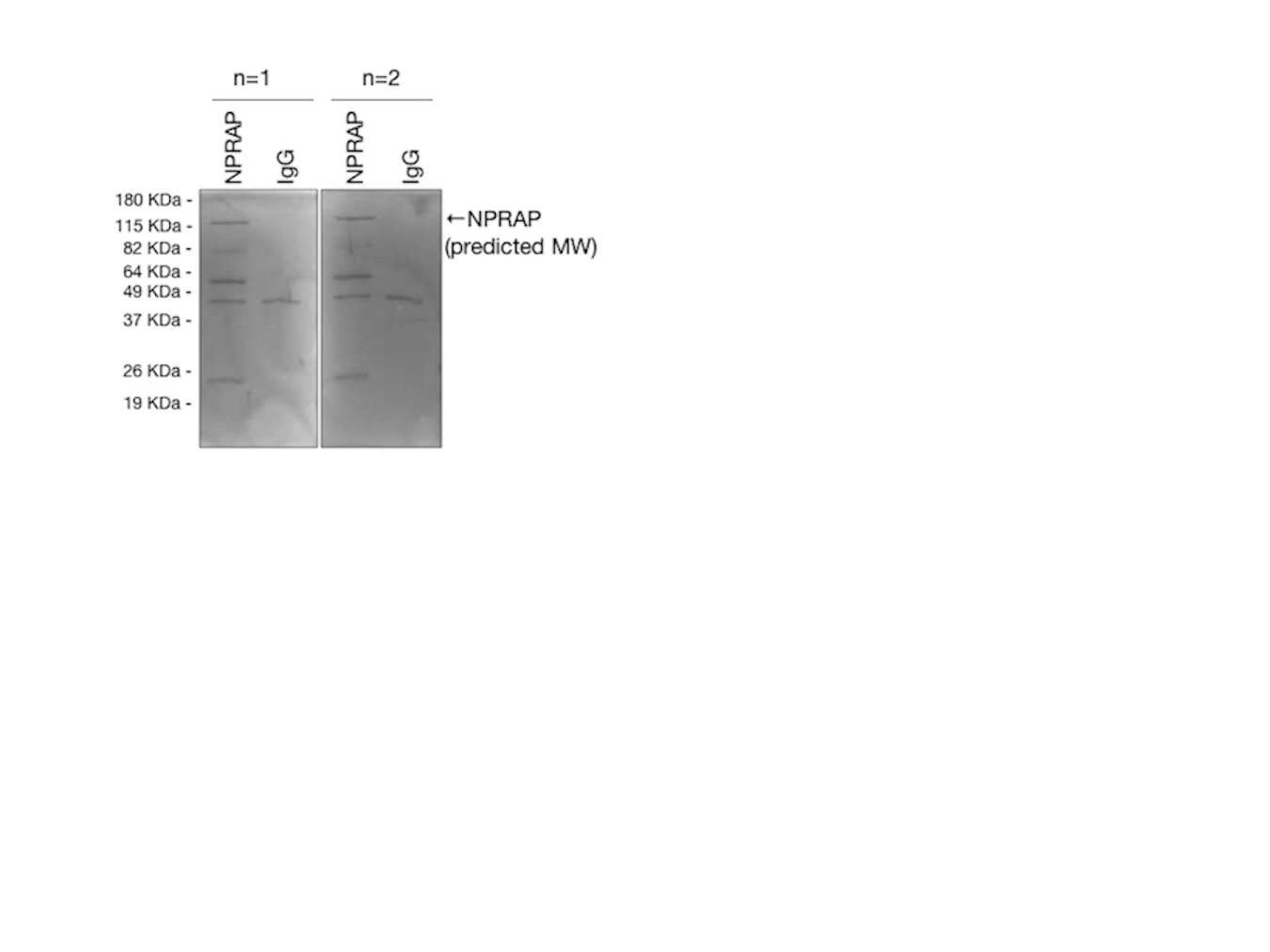

Supplement: Figure S1 — Coomassie stain patterns for protein samples from cells overexpressing NPRAP (lanes 2 and 4). In lanes 3 and 5, the correspondent patterns for mouse serum IgG controls. Protein tracks from these gels were excised and further analyzed by LC MS/MS as described in the materials and methods section. MW: molecular weight. (TIFF) [file pone.0025379.s001.tiff]

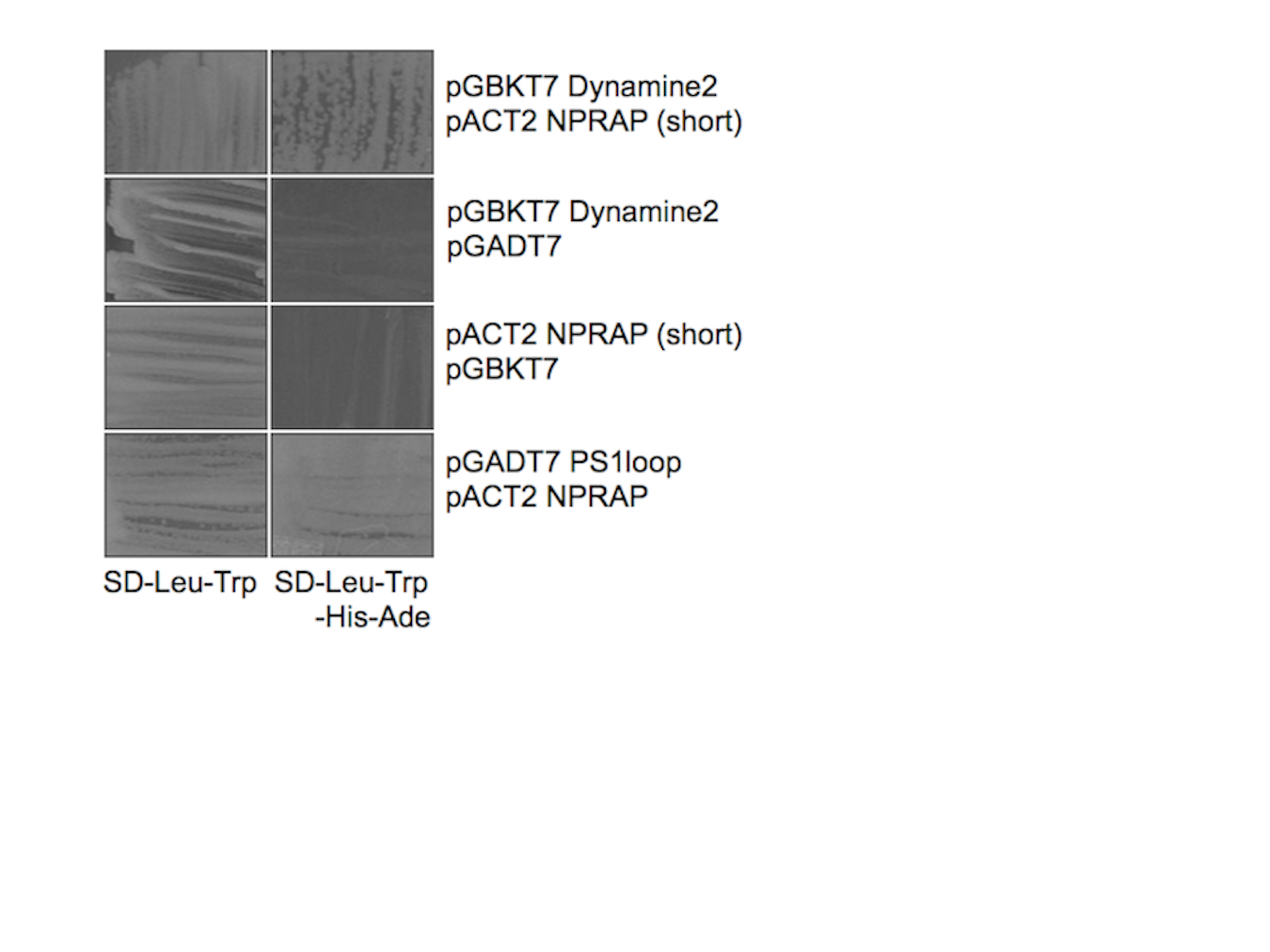

Supplement: Figure S2 — NPRAP binding to dynamin 2. A shorter NPRAP clone beginning in its fifth arm repeat (amino acid 650) also interacts directly with dynamin 2, strongly suggesting that their binding site is located after that repeat and within NPRAP's C-terminal sequence. (TIFF) [file pone.0025379.s002.tiff]
